# Psychosis associated with cannabis withdrawal: systematic review and case series

**DOI:** 10.1192/bjp.2024.175

**Published:** 2025-05

**Authors:** Edward Chesney, Thomas J. Reilly, Fraser Scott, Ikram Slimani, Ananya Sarma, Daisy Kornblum, Dominic Oliver, Philip McGuire

**Affiliations:** Department of Addictions, King's College London, UK; Department of Psychosis Studies, King's College London, UK; South London and Maudsley NHS Foundation Trust, London, UK; Department of Psychiatry, University of Oxford, UK; NIHR Oxford Health Biomedical Research Centre, Oxford, UK

**Keywords:** Cannabis dependence, substance withdrawal syndrome, psychosis and first-episode psychosis, case series, systematic review

## Abstract

**Background:**

Abrupt cessation of heavy cannabis use can cause a withdrawal syndrome characterised by irritability, anxiety, insomnia, reduced appetite and restlessness. Recent reports have also described people in whom cannabis withdrawal immediately preceded the acute onset of psychosis.

**Aims:**

To identify cases of psychosis associated with cannabis withdrawal.

**Method:**

We completed a systematic review of the literature, which comprised case reports, case series and other studies. We also searched a large electronic database of psychiatric healthcare records.

**Results:**

The systematic review identified 44 individuals from 21 studies in whom cannabis withdrawal preceded the development of acute psychosis. In the health record study, we identified another 68 people, of whom 47 involved a first episode of psychosis and 21 represented further episodes of an existing psychotic disorder. Almost all people were daily cannabis users who had stopped using cannabis abruptly. Individuals who continued to use cannabis after the acute psychotic episode had a much higher risk of subsequent relapse than those who abstained (odds ratio 13.9 [95% CI: 4.1 to 56.9]; *χ*^2^ = 20.1, *P* < 0.00001).

**Conclusions:**

Abrupt cannabis withdrawal may act as a trigger for the first episode of psychosis and a relapse of an existing psychosis. Acute psychotic symptoms can emerge after the cessation, as well as following the use, of cannabis.

Cannabis use is associated with an increased risk of developing psychosis, especially with daily use of high-potency cannabis.^1–3^ A recent meta-analysis indicates that among individuals who develop a first episode of psychosis, 36% also meet diagnostic criteria for a cannabis use disorder.^4^ After psychosis onset, continued cannabis use is associated with symptom exacerbation, a higher risk of relapse and reductions in level of functioning and quality of life.^5–7^ These adverse effects are particularly evident in heavy users.^7,8^ Both recreational cannabis use and the prevalence of cannabis use disorders in the general population are increasing,^9,10^ a trend which may be related to the legalisation and decriminalisation of cannabis use in some countries, such as the USA and Canada.^10,11^ In Norway, Denmark and Sweden, between 2000 and 2016, the incidence of ‘cannabis-induced psychosis’ rose by 67, 115 and 238%, respectively.^12^ In Canada between 2016 and 2021, the rate of hospitalisations for ‘cannabis-induced psychosis’ increased by 40%.^13^

Both the intoxicating (euphoria, relaxation) and the adverse effects of cannabis (anxiety, cognitive impairment and paranoia) are attributable to its main psychoactive constituent, delta-9-tetrahydrocannabinol (THC).^14,15^ A proportion of cannabis users develop a cannabis use disorder, which is characterised by increased use of the drug, psychosocial impairment and physiological changes, with increased cannabis tolerance and a withdrawal syndrome.^16^ According to DSM-5, the latter requires the presence of at least three of the following seven symptoms: (a) irritability, anger or aggression; (b) nervousness or anxiety; (c) sleep difficulty (e.g. insomnia, disturbing dreams); (d) decreased appetite or weight loss; (e) restlessness; (f) depressed mood; and (g) physical symptoms, including abdominal pain, shakiness/tremors, sweating, fever, chills or headache, which cause significant discomfort.^17^ The criteria also state that these symptoms should emerge within approximately one week of cessation, and after heavy and prolonged use of cannabis. They typically begin within 24–48 h, peak after 2–6 days and subside after 1–2 weeks. In a systematic review of studies including people with regular or dependent use of cannabinoids, the prevalence of cannabis withdrawal syndrome was 17% in population-based samples, 54% in out-patient samples and 87% in in-patient samples.^18^

Recently, there have been case reports of acute psychosis developing in the context of cannabis withdrawal.^19–21^ In the present study, we examined this association by reviewing all studies in the published literature, and by using a large registry of electronic health records to identify our own patient sample. The primary aim of our study was to characterise the clinical characteristics of cannabis withdrawal-associated psychosis.

## Method

### Systematic review

The systematic review was registered on the International Prospective Register of Systematic Reviews (PROSPERO; CRD42022378512).

### Eligibility

Studies describing cases of psychosis or mania triggered by acute withdrawal of cannabis or synthetic cannabinoids were included. In addition to identifying case reports and case series, we included reports from other studies such as case-control, cohort and controlled trials. We did not exclude letters, conference abstracts or posters.

### Search strategy

PsycINFO, Embase and MEDLINE were searched from the date of inception until 12 June 2024. The initial search was completed on 8 December 2022 and was updated on 13 June 2024. For all databases, we used the following terms to search titles, abstracts and keywords: (cannabis OR marijuana OR marihuana OR tetrahydrocannabinol OR THC OR cannabinoid*) AND (psychosis OR psychotic OR schizophrenia OR schizophreniform OR schizoaffective OR mania OR bipolar OR hallucinat* OR delusio* OR paranoi*) AND (withdrawal OR discontinuation OR cessation). Additional targeted searches were completed using Google Scholar, and the references of included articles were reviewed to identify additional studies.

Two reviewers (from E.C., A.S. and I.S.) independently screened the titles and then abstracts of all articles identified by the search. The full texts of the selected articles were subsequently reviewed by two independent authors (A.S. and I.S.). Any disagreements were resolved after discussion with a third author (E.C.).

### Data extraction and synthesis

Outcome data were independently extracted by two authors (A.S. and I.S.). Data included demographics, and past psychiatric, family and substance use history. Information regarding cannabis withdrawal-associated psychosis included the rate of discontinuation of cannabis use, cannabis withdrawal symptoms, time from cannabis discontinuation to onset of psychosis, psychosis symptoms, clinical management and prognosis. The two sets of extracted data were compared, and any differences were resolved by a third author (E.C.). Data extraction was started on 24 December 2022 and completed on 13 June 2024.

### Cases identified from electronic health records

#### Setting and search strategy

To identify cases of cannabis withdrawal-associated psychosis, we used the South London and Maudsley NHS Foundation Trust Biomedical Research Centre Case Register, which contains anonymised records of over 400 000 patients receiving secondary mental healthcare in South East London. This was searched using the Clinical Record Interactive Search (CRIS) system in April 2023.^22^ CRIS received ethical approval as an anonymised data-set for secondary analyses from Oxfordshire Research Ethics Committee C (ref: 23/SC/0257).

We searched for patients with clinical entries (including emergency assessments, progress notes, ward rounds, clinic letters, discharge summaries and structured diagnostic entries) that contained terms that we selected as being related to both cannabis withdrawal and to psychosis. The cannabis withdrawal terms included: ‘cannabis withdrawal’, ‘withdrawal $ cannabis’ and ‘stopped $ cannabis $ days’, where ‘$’ represents a ‘wildcard’. The psychosis terms included ‘psychosis’, ‘psychotic’, ‘schizophrenia’, ‘bipolar’ and ‘mania’. A full list of the terms used is provided in the supplementary materials. We also searched for patients with a psychosis-related diagnosis according to ICD-10 criteria, including: organic mental disorders (F0x); mental and behavioural disorders due to use of cannabinoids, psychotic disorder (F12.5), schizophrenia spectrum disorders (F20-29), mania and bipolar affective disorder (F30-31), and severe depression with psychotic symptoms (F32.3; F33.3), who also had a term for cannabis withdrawal in a clinical entry.

#### Eligibility

First, each clinical entry was screened by a psychiatrist (E.C.). Possible cases were then reviewed by two psychiatrists (from E.C., F.S., T.R.) and were included if both agreed that the patient had an episode of psychosis which was ‘probably’ or ‘definitely’ associated with cannabis withdrawal. Manic psychoses and those associated with bipolar affective disorder or major depression were included. Cases where the psychotic symptoms were transient (i.e. <7 days) were excluded, as this is the minimum duration of symptoms required to meet the diagnostic criteria for first episode psychosis.^23^ Cases with comorbid substance use or alcohol use disorders were not excluded, as long as cannabis withdrawal was determined to be the primary trigger of the episode of illness. Disagreements were adjudicated by a third psychiatrist.

#### Data extraction

Clinical records, including emergency assessments, progress notes, ward rounds, clinic letters and discharge summaries, were reviewed by researchers (E.C., F.S., T.R., A.S., I.S.). To identify psychotic symptoms during the first 30 days after presentation with acute psychosis, we used the Operational Criteria in Studies of Psychotic Illness (OPCRIT) symptom checklist.^24^ DSM-5 cannabis withdrawal symptoms, reported within the first 14 days after cannabis cessation, were also extracted. We recorded the presence or absence of symptoms but not their severity. All demographic and clinical data were either extracted or reviewed by a psychiatrist, and symptom data were extracted by psychiatrists only (E.C., F.S., T.R.). Secondary diagnoses of alcohol and substance use diagnoses were determined by psychiatrists according to clinical judgement. To assess the quality of data extraction, OPCRIT data were extracted for 32 cases by a second psychiatrist; the inter-rater reliability (Cohen's kappa) was 0.96. Relapse was defined as admission to psychiatric hospital, home treatment team or intensive community management.

### Statistical analysis

Continuous outcomes were reported as means with standard deviation (s.d.), or medians with interquartile range (IQR). Categorical outcomes were reported as frequencies. The relationship between the number of cases per year and year of presentation was analysed with a Spearman's rank correlation. Psychosis relapse was analysed according to cannabis use trajectory (abstinence versus persistent use) with a Chi-squared test. The threshold for statistical significance was *P* < 0.05. Inter-rater reliability was estimated using percentage agreement and by calculating Cohen's kappa. All analyses were completed using R version 4.2.2 for Mac OS Sonoma 14.6.1.

## Results

### Study 1: systematic review

We identified 1635 studies (Supplementary Fig. 1 available at https://doi.org/10.1192/bjp.2024.175), of which 21 were included in the present review. These included 12 case reports^19–21,25–33^ and 22 cases described in five case series^34–38^ (Table 1). An additional ten cases were identified in one controlled trial^39^ and three other studies.^40–42^ Collectively, these studies comprised a total of 44 cases.

**Table 1:** Demographic and clinical features of cases of cannabis withdrawal-associated psychosis identified by the systematic review

Author	Number of cases	Age and gender	Past psychiatric and family history	Current cannabis use	Onset	Withdrawal symptoms	Psychosis symptoms	Treatment and outcome
Fraser et al 1949^34^	9	Males^a^	Not reported	Continuous use for ‘some years’	Not clear	Cannabis craving, appetite changes, irritability, depressed mood	Violence, extreme irritability, paranoia, excitement, logorrhoea, argumentativeness, disinhibited behaviour, poor self-care, auditory and visual hallucinations	Sedation with barbitone or paraldehyde. All recovered after 3–6 weeks, though were weak and emaciated
Rohr et al 1989^35^	1	25, M	None	7 g/day for 5 years	Deterioration over 1 week	Anorexia, sleep disturbance, tremors, cravings. Day 4 of hospitalisation: attempted suicide by hanging	Thought disorder, disorientation, agitation, paranoia, mood swings, visual, auditory and tactile hallucinations	‘Much improved’ after 9 days
Redvers et al 2004^25^	1	42, M	Six psychotic episodes associated with cannabis abstinence	Not reported	1–2 weeks	Insomnia, vivid dreams, restlessness	Fearfulness, persecutory delusions, delusions of reference, aggression, violence, thought block and depersonalisation with subsequent self-harm	Symptoms resolved with antipsychotic medication. Asymptomatic after using cannabis again
Yazici et al 2017^36^	1	26, M	Two hospitalisations related to substance use	Intense synthetic cannabinoid use for 1 week prior to deterioration	2 days	Nervous, irritable, insomnia, aggression	Disorientation to place and time, disorganised behaviour, euphoria, irritable mood, logorrhoea, persecutory delusions, auditory and visual hallucinations, and impaired insight	Improved after 2 days of antipsychotic medication
Carson et al 2018^26^	1	20, F	Anxiety	Daily use for six years	4 days after ‘abrupt’ discontinuation	Extreme anxiety, restlessness, sweating	Religious preoccupation. Referencing a biblical passage, she amputated her right hand with a kitchen knife. Paranoia	Aripiprazole, trazodone and clonazepam led to complete remission of symptoms
Joseph et al 2018^27^	1	23, F	Depression, post-traumatic stress disorder, two suicide attempts. Mother had bipolar affective disorder	Three blunts at least 3x/day for 4 years	2 days after ‘abrupt’ discontinuation	Not reported	Paranoid delusions, auditory hallucinations, racing thoughts and pacing	Improved after 2 days. Risperidone was discontinued at discharge
Shilpakar et al 2019^28^	1	37, F	Not reported	9–10 joints/day for 2 years	4 days after ‘sudden’ discontinuation	Not reported	Paranoid and unusual behaviour, aggressive and argumentative; ‘manic symptoms’	Sodium valproate and antipsychotic medication. Two more hospitalisations within three months. Diagnosed with bipolar affective disorder
Roy et al 2020^29^	1	22, M	None	4–5 chillams of ganja and 2–3 pellets of bhang almost daily	1 day after ‘abrupt’ discontinuation	Insomnia, irritability, anger, anxiety	Suspiciousness and auditory hallucinations	Significant improvement after 2 weeks of carbamazepine, risperidone and lorazepam
Doyle et al 2020^30^	1	15, M	Depression and anxiety	3 g/day for 1 year	1 day	‘Profound’ insomnia and poor appetite. Anxiety, depression, suicidal thoughts and gestures, agitation and aggression	Guarded, paranoid, disorganised, evasive, and demanding to leave. Within 72 h, he started experiencing auditory/visual hallucinations with paranoia, disorganised thoughts and behaviour	Dramatic’ improvement after day 3, following sleep. Treatment with mirtazapine, haloperidol quetiapine, trazodone, olanzapine, lorazepam and paroxetine
Marin et al 2021^31^	1	29, M	None	2.5 g/day	Over the following weeks	Irritability, nervousness, insomnia, depressed mood, apathy and decreased appetite	Fearful and persecutory delusions. Brief psychiatric rating scale score 72 (severely ill)	Complete remission of symptoms after 7 days treatment with olanzapine and lorazepam
Shakya et al 2021^21^	1	20, M	Uncle had a history of psychosis	‘High most of the time’	3 days	Restlessness, headache, abdominal pain, decrease in appetite, irritability, aggression, difficulty sleeping, craving	Social isolation, racing thoughts, muttering to himself, suspiciousness, delusions of reference, persecutory delusions, loss of insight	Antipsychotics. Complete remission after 36 h
MacCamy et al 2021^32^	1	44, M	A similar episode in his 20s requiring in-patient psychiatric hospitalisation	Heavy use for 30 years	42 days, abruptly	Insomnia, bilateral tremors	Confabulation, visual hallucinations	Treatment with diphenhydramine, haloperidol, hydroxyzine, lorazepam, midazolam, olanzapine, quetiapine and IV dexmedetomidine in intensive care. Discharged on day 15
Kung et al 2022^20^	1	25, M	Not reported	Continuous use for 2 years	10 days	Not reported	Persecutory delusions, auditory hallucinations with commanding voices, visual hallucinations. He attempted suicide by cutting his wrist to ‘escape from the persecution’	Treated with brexpiprazole. Symptoms resolved after 5 days
Ramos et al 2022^19^	1	32, F	Previous episode of psychosis associated with cannabis withdrawal	2 g/day for 15 years	Symptoms started after 2 days, disorganised behaviour by 1 week	Insomnia, irritability	Dishevelled, with poor hygiene, agitated, restless, aggressive, unpleasant and uncooperative, extremely irritable, with persecutory delusions, disorganised speech. No hallucinations or manic symptoms. Loss of insight	Gradual improvement over 1 week with aripiprazole and diazepam. She was maintained on antipsychotic medication and restarted using cannabis at a lower dose (about 0.5 g per day)
Villa et al 2023^33^	1	35, M	Not reported	1 g/day for 14 months	4–5 days	Insomnia	Mental hyper-clarity, flight of ideas, insomnia, inadequacy, hyperactivity and increased energy, hypersexuality and euphoria	Hospitalisation
Cohen et al 2024^37^	8	Mean age 25; 7/8 cases male	Four cases had no past psychiatric history. Three had a history of psychosis, of whom two had a history of psychosis or suicidality after cannabis cessation	1 to 4 g/day (*n* = 4)	6–7 days (*n* = 6), 20 days (*n* = 1), unknown (*n* = 1)	All cases met criteria for DSM-5 cannabis withdrawal syndrome	A broad range of symptoms were described, including paranoia, persecutory delusions, grandiosity, hallucinations, thought disorder and catatonic symptoms. The mean retrospective Positive and Negative Syndrome Scale positive subscale score was 23 (range 14 to 37)	All patients were treated with antipsychotic medication and were hospitalised from between 3 and 28 days
Salmerón et al 2024^38^	3	22, M, 19, M and 20, M	None significant	Problematic use since age 16 5–10 units/day Cannabis dependence since age 15	15 days; 6 weeks; and 1 month	Insomnia, irritability, verbal aggression, agitation, mood instability	Increased energy, euphoria, pressured speech, incoherent speech, disorganised behaviour, paranoid, grandiose and religious delusions, loss of insight	Lithium, paliperidone and olanzapine; in hospital for 10 days Lithium and aripiprazole; in hospital for 14 days Lithium, olanzapine, paliperidone and zuclopenthixol, and then ECT (11 sessions); in hospital for 56 days

a. The age of participants was not reported in the original study.

The two earliest reports were published in the 1940s, one of which was by a doctor in the British Army responsible for Indian troops serving in the Asia-Pacific theatre who were unable to access the ‘Indian hemp plant’ while on campaign.^34^ The second was from a controlled trial of a synthetic THC homologue, ‘pyrahexyl’.^39^ Two other studies described cases associated with withdrawal from synthetic cannabinoids.^36,40^

Of the 34 people described in case series and case reports, most (*n* = 29; 85.3%) were male, with a mean age of 26.2 years (s.d.: 8.6). Most had started using cannabis in early adolescence, were heavy cannabis users (i.e. at least 1 g/day) and had abruptly stopped using cannabis. The median time from discontinuation of cannabis use to the emergence of psychosis symptoms was 6 days. Thirteen individuals reported sleep disturbance, which was often noted as being severe or ‘profound’. Irritability and anxiety were also common. Most people reported psychotic symptoms such as delusions, hallucinations and disorganised behaviour, and almost all required antipsychotic medications and in-patient admission. At least nine people presented with manic symptoms.^19,28,33,36–38^ Two individuals severed their wrists,^20,26^ two attempted suicide^35,38^ and two reported suicidal ideation.^30,37^ One individual was diagnosed with bipolar affective disorder after two subsequent psychosis relapses.^28^ Six people had a past history of psychotic illness, of which four had previous episodes following cannabis withdrawal: three had a single previous episode associated with cannabis withdrawal,^19,32,37^ and the other had experienced six previous episodes associated with it.^25^

Our systematic search identified four additional studies with a total of ten individuals. Williams and colleagues described a controlled trial where six male prisoners were administered pyrahexyl, a synthetic homologue of THC, for between 26 and 31 days.^39^ Participants were allowed to choose their own dose, and by the end of the study the average dose was about 1600 mg/day. After abrupt discontinuation of treatment, two participants demonstrated noteworthy reactions after three days. One had a ‘panic reaction’, while the other had a hypomanic reaction, characterised by overactivity, euphoria and increased psychomotor activity, which resolved after eight days. Bernhardson and colleagues described 46 of the first cases of cannabis-associated psychosis in Sweden between 1966 and 1970.^41^ They noted that two of these people developed psychosis after 1–2 weeks of abstinence. Skryabin and colleagues described 60 cases of individuals with psychosis associated with synthetic cannabinoids in Russia.^40^ They noted that four people developed a psychotic disorder ‘in the context of the withdrawal syndrome’, with symptoms such as visual hallucinations and persecutory delusions. Teitel reported another three cases associated with a ‘manic-depressive type of illness’.^42^

### Study 2: cases from electronic records

The search identified 1058 people, of whom 240 were identified as potential cases for further assessment. Of these, 68 were assessed by two psychiatrists as having ‘probably’ or ‘definitely’ a case of cannabis withdrawal-associated psychosis. Of these, 47 were associated with a first episode of psychosis, and 21 involved a relapse of psychosis in individuals with a history of a psychotic disorder. Most of the assessments (*n* = 39; 57.4%) relied on the participant's self-report alone. In 18 cases (26.5%), there was also collateral information from a friend or family member to corroborate the history. In 11 cases (16.2%), there was a contemporaneous clinical report that documented that the cessation of cannabis use occurred prior to the onset of psychosis.

Demographic and clinical characteristics of each group are described in Table 2. The sample was predominantly male (*n* = 56; 82.4%), identified with Black and minority ethnic groups (*n* = 41; 60.3%) and had relatively few comorbid alcohol or substance use disorders (*n* = 10; 14.7%). The mean age at onset for the first episode group was 26.4 years, which is similar to that reported in previous studies of cannabis users with psychosis.^2^ The earliest episode was identified in 2004; the most recent in 2023. There was a positive correlation between year of presentation and number of cases per year (Spearman's rho = 0.68; *P* = 0.0002).

**Table 2:** Demographic and clinical characteristics of 68 cases of cannabis withdrawal-associated psychosis

Characteristic	All cases	First episode	Relapse
Total sample (*n*)	68	47	21
Age at index episode (years)	27.3 (9.4)	26.4 (9.2)	29.4 (9.7)
Gender			
Male	56 (82.4%)	41 (87.2%)	15 (71.4%)
Female	12 (17.6%)	6 (12.8%)	6 (28.6%)
Ethnicity			
White	27 (39.7%)	16 (34.0%)	11 (52.4%)
Black	24 (35.3%)	18 (38.3%)	6 (28.6%)
Mixed or Other	17 (25.0%)	13 (27.7%)	4 (19.0%)
Family history of psychosis			
First degree	10 (14.7%)	4 (8.5%)	6 (28.6%)
Second degree	11 (16.2%)	8 (17.0%)	3 (14.3%)
None	47 (69.1%)	35 (74.5%)	12 (57.1%)
Comorbid substance use disorder (current)			
Any (excluding cannabis)	10 (14.7%)	6 (12.8%)	4 (19.0%)
Alcohol	6 (8.8%)	4 (8.5%)	2 (9.5%)
Cocaine	2 (2.9%)	1 (2.1%)	1 (4.8%)
Other	3 (4.4%)	2 (4.3%)	1 (4.8%)
Diagnosis at follow-up (ICD-10)			
Substance use disorder (F12, F19)	9 (13.2%)	7 (14.9%)	2 (9.5%)
Schizophrenia, schizotypal and delusional disorders (F20–F29)	39 (57.4%)	27 (57.4%)	12 (57.1%)
Manic episode or Bipolar affective disorder (F30, F31)	10 (14.7%)	5 (10.6%)	5 (23.8%)
Not specified/Other	10 (14.7%)	8 (17.0%)	2 (9.5%)

#### Cannabis use histories

The quantity and quality of the clinical data describing the intensity and frequency of cannabis use were highly variable. The only reported method of cannabis use was smoking. The health records indicated that most people (*n* = 59; 86.8%) were daily users, and a minority (*n* = 8; 11.8%) were ‘regular’ or ‘frequent’ users. In one case (1.5%), there was no information regarding frequency of use. Of the 50 individuals for whom information on cannabis potency was available, most (*n* = 35; 70.0%) used high potency cannabis, with the remainder using more than one type (*n* = 6; 12.0%), hash (*n* = 4; 8.0%) or low potency cannabis (*n* = 5; 10.0%). Among those for whom there were data on the number of joints used per day (*n* = 34), the median number was 5 (IQR: 3–6.5). Information on the amount of cannabis used was only available in a small number of cases (*n* = 13). The median amount was 3 g/day (IQR: 2–5.5). Age at first cannabis use was reported in 51 cases, and the mean was 16.1 years (s.d.: 4.1).

#### Cannabis withdrawal: symptoms and time until emergence of frank psychosis

**Fig. 1:**
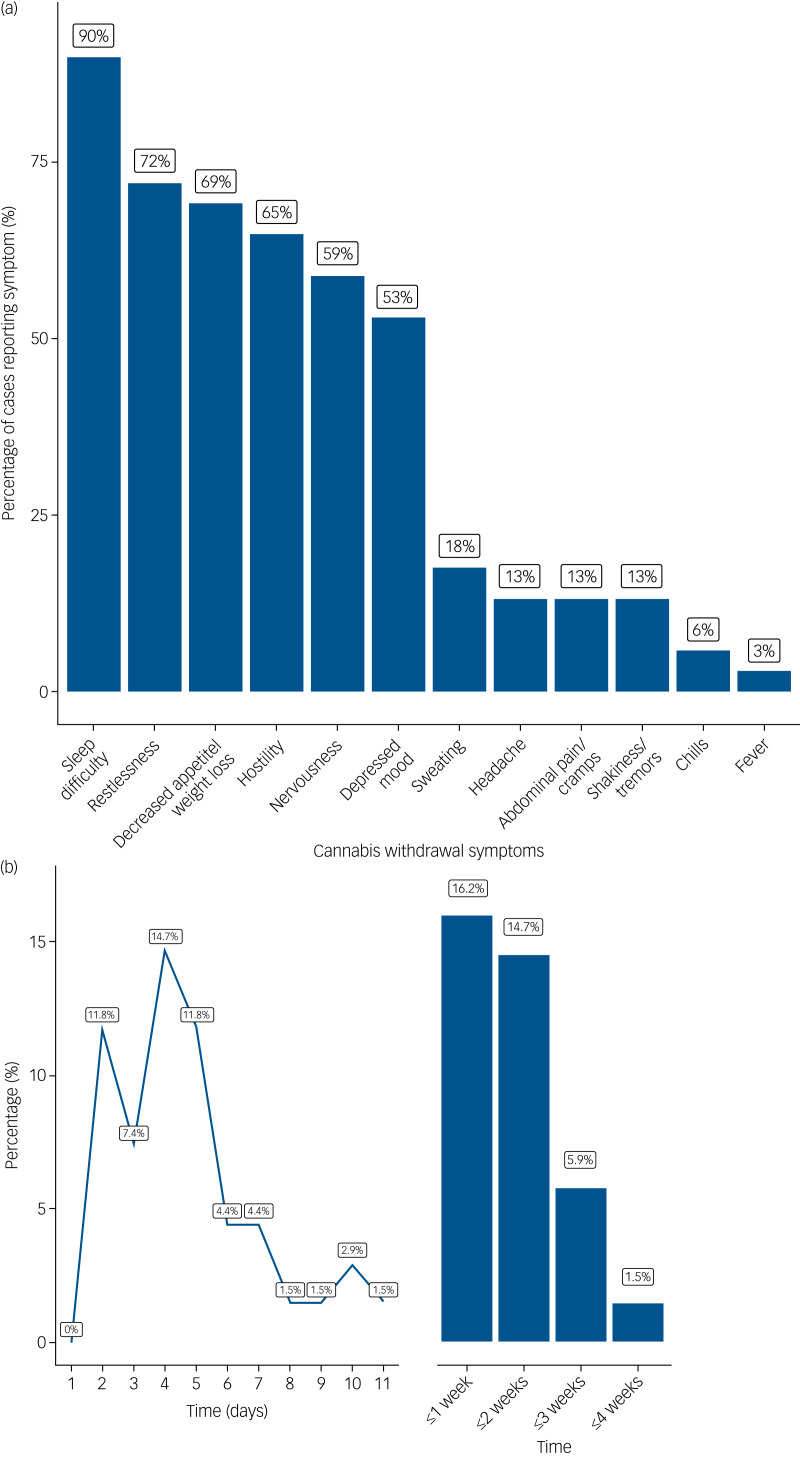
(a) Proportion of cases reporting individual symptoms of cannabis withdrawal. (b) Time from cessation of cannabis use to presentation with psychosis.

The majority of participants (*n* = 55; 80.9%) had stopped using cannabis abruptly. Two people (2.9%) had reduced their use before stopping completely, and two (2.9%) had reduced their use substantially but not stopped. In nine participants (13.2%) there was insufficient data to characterise the rate of cessation. The most common withdrawal symptoms were sleep difficulty (89.7% of cases), restlessness (72.1%), reduced appetite/weight (69.1%) and hostility (64.7%; Fig. 1(a)). Almost half of the participants (46.5%) reported at least one somatic symptom. Most people (63; 92.6%) had at least three withdrawal symptoms and therefore met the symptomatic criteria for DSM-5 cannabis withdrawal syndrome. However, terms describing cannabis withdrawal or the cannabis withdrawal syndrome were only written in the clinical records in a minority of cases (24; 35.3%). When cannabis withdrawal was mentioned, it was more likely to have been done for cases involving a relapse of psychosis than for a first episode of psychosis (relapse: *n* = 11/21 [52.4%], FEP: *n* = 13/47 [27.7%] *χ*^2^ = 3.88, *P* = 0.049). The time from discontinuation of cannabis use to the emergence of frank psychosis ranged from 2 days to 4 weeks (Fig. 1(b)). Psychosis emerged within 1 week of cessation in 48 (70.6%) of cases, with the highest incidence at 4 days post-cessation.

#### Symptoms, risk, clinical management and prognosis

**Fig. 2:**
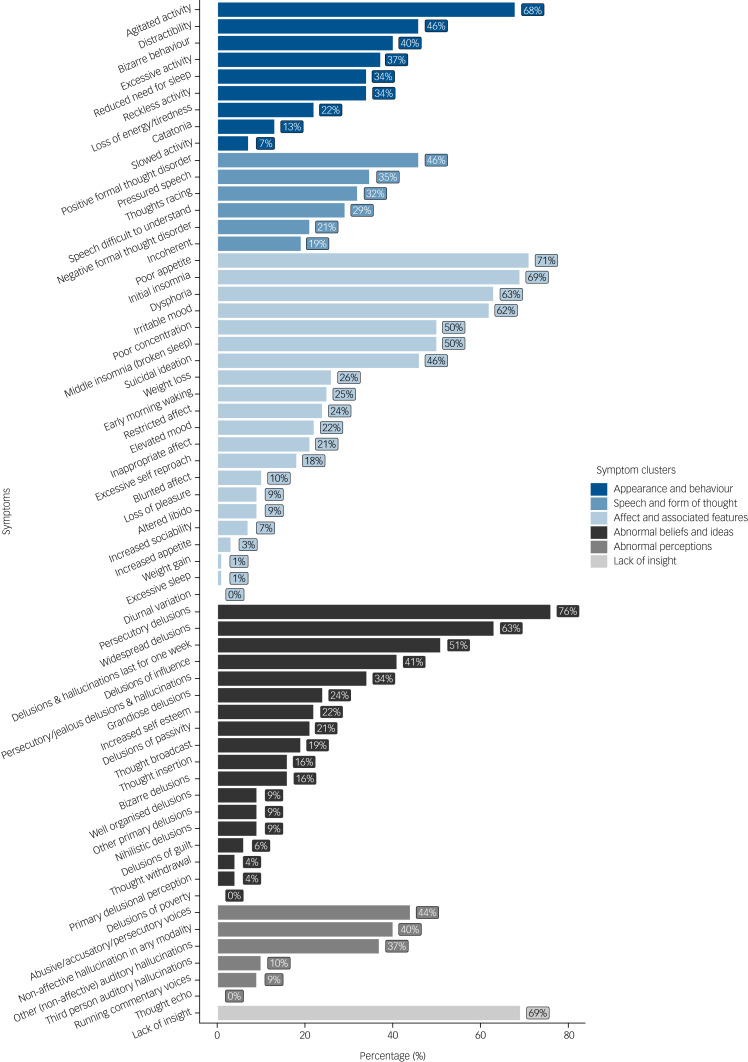
Proportion of cases with Operational Criteria in Studies of Psychotic Illness (OPCRIT) psychosis symptoms recorded in health records.

Psychotic symptoms were assessed by applying OPCRIT criteria to the health record data (Fig. 2). The symptoms with the highest prevalence were persecutory delusions (76.5%), poor appetite (70.6%), initial insomnia (69.1%), loss of insight (69.1%) and agitated activity (67.6%). A sleep disturbance of any type was identified in 61 (89.7%) of participants. In many people it was noted to be severe and persistent, with example descriptions including: ‘absolutely no sleep for one week’, ‘has not slept for 72 h’, ‘little or no sleep over the last seven days’ and ‘slept for only two hours’.

Twenty-eight individuals presented significant risks of harm to self or others, including: severe self-mutilation or suicide attempt (*n* = 3), serious threats or plans of suicide (*n* = 3) or command hallucinations to harm self (*n* = 3); attempted murder (*n* = 2), threats to kill (*n* = 3), physical violence to others (*n* = 8), commands to harm others (*n* = 2) or serious threats of violence to others (*n* = 2); fire-setting (*n* = 2) or other extremely reckless behaviour (*n* = 2). Several people presented more than one significant risk.

Regarding immediate clinical management, one person (1.5%) was referred to primary care, nine (13.2%) were managed by out-patient mental health services, seven (10.3%) received intensive home treatment, and 51 (75.0%) were admitted to psychiatric hospital, of whom five (7.4%) required intensive care, and one (1.5%) was managed in a secure forensic hospital. Of the 51 people admitted to hospital, 40 (78.4%) were detained involuntarily. Most individuals (*n* = 61, 89.7%) were prescribed antipsychotic medication. One person (1.5%) was prescribed quetiapine at a sub-antipsychotic dose, and two (2.9%) received mood stabilising medication only. Two people (2.9%) were managed with sedative medication only, and two (2.9%) were managed without medication.

In 60 people there was information describing their subsequent clinical course and use of cannabis. Most individuals (38; 63.3%) continued to use cannabis, and most of this subgroup (31; 81.6%) experienced a relapse of psychosis. A minority (22; 36.7%) abstained from cannabis after discharge or recovery, and the relapse rate in this subgroup was 22.7% (*n* = 5). The risk of relapse in those who continued to use cannabis was much higher than in those who abstained (odds ratio 13.9 [95% CI: 4.1 to 56.9]; *χ*^2^ = 20.1, *P* < 0.00001). When the analysis was limited to first-episode cases (*n* = 42), 25 (60.9%) continued to use cannabis, of whom 21 (84.0%) relapsed. Of the 17 people who abstained from cannabis, only four (23.5%) relapsed. Again, those who continued to use cannabis had a much higher risk of relapse than those who abstained (odds ratio = 15.1 [95% CI: 3.5 to 84.8]; *χ*^2^ = 15.4, *P* < 0.0001).

## Discussion

We identified a total of 68 cases of psychosis associated with cannabis withdrawal in a large database of mental health records. Cannabis withdrawal was often associated with a first episode of psychosis, but there were also cases of a psychotic relapse of a pre-existing psychotic disorder. Most individuals were male, used cannabis daily, stopped using cannabis abruptly and developed psychosis within a week of cessation. Most had symptoms consistent with the DSM-5 criteria for cannabis withdrawal syndrome, with sleep disturbance the most commonly reported symptom. After recovery from the psychosis, the risk of relapse was much higher in those who continued to use cannabis than in those who stopped.

We also conducted the first systematic review of cannabis withdrawal-associated psychosis. This identified a total of 44 cases from 21 reports published over the last 80 years. The demographic and clinical features of these cases were similar to those identified from the health record search, with a high proportion of male individuals (higher than is observed in people with cannabis use disorder^43^), frequent histories of heavy cannabis use followed by abrupt withdrawal, and a history of insomnia prior to psychosis onset.

In our health record study, although most people appeared to meet the symptomatic criteria for a diagnosis of cannabis withdrawal syndrome, the term ‘cannabis withdrawal’ (or an equivalent term) was often not recorded by the treating clinicians. When cannabis withdrawal was mentioned, this was more likely when withdrawal was associated with a relapse of a pre-existing disorder, as opposed to a first episode of psychosis. This may reflect the availability of prior clinical assessments which demonstrated clinical stability despite ongoing cannabis use, and deterioration in mental state only after cessation. Our methods for identifying individuals for our case series were reliant on the notes recorded by clinicians, and the under-recognition of cannabis withdrawal syndrome by clinicians suggests that more people may have been identified if there had been more awareness of this as a clinical entity. Other potentially contributing factors are a reluctance of people to disclose information about cannabis use, and an inability to do this when severely unwell.^44^

Little is known about the molecular mechanisms that might underlie the development of psychosis secondary to cannabis withdrawal. However, it might involve similar pathways to those that mediate the acute effects of THC on psychosis. Positron emission tomography and single-photon emission computed tomography studies in healthy volunteers have found only small changes in striatal dopamine release following administration of THC^45–47^ However, studies in heavy and dependent cannabis users have reported reductions in striatal dopamine synthesis capacity and release,^48–50^ which are the opposite to the changes in dopamine function seen in people with a psychotic disorder.^51^ The induction of transient psychotic symptoms in healthy volunteers following administration of THC is correlated with its effects on activation in the medial temporal cortex and the striatum,^52^ brain regions that are thought to be critical to the pathophysiology of psychotic disorders.^53^ The extent to which psychosis following cannabis withdrawal is related to functional changes in these areas has yet to be investigated.

Howard and colleagues used OPCRIT to describe the psychopathology of 336 people with psychosis independent of cannabis use or withdrawal.^54^ Compared with our individuals with cannabis withdrawal, these people were less likely to have positive (27.4% versus 45.6%) or negative thought disorder (11.0% versus 20.6%), restricted affect (13.1% versus 23.5%) and catatonia (8.0% versus 13.2%), but more likely to have persecutory delusions with hallucinations (58.6% versus 33.8%), third-person auditory hallucinations (28.9% versus 10.3%) and thought withdrawal (12.5% versus 4.4%). The Howard et al study did not report the number of people with sleep disturbance or affective symptoms, but these symptoms were assessed using OPCRIT in another study in people with bipolar affective disorder or schizophrenia, independent of cannabis use or withdrawal.^55^ This study found that insomnia, agitation, and appetite or weight changes were much less common in people with schizophrenia (28.2, 28.8 and 12.9%, respectively) than in our series (89.7, 67.6 and 75.0%), but were evident to a similar extent in people with bipolar disorder (95.5, 74.8 and 87.3%, respectively). As only a small proportion of our individuals (14.7%) had a diagnosis of mania or bipolar affective disorder, this suggests that these symptoms are associated with psychosis following cannabis withdrawal, rather than being features of an affective psychosis.

In both of our studies, most of the people experienced insomnia prior to the emergence of psychosis, and the insomnia was often severe or prolonged. Sleep disturbance is a common complaint in people with psychosis, and often predates psychotic relapse by 1–2 weeks, independent of cannabis use or withdrawal.^56^ It is also common in individuals who are at clinical high risk of developing psychosis^57^ and has been used as a factor in a prediction model of transition to psychosis.^58^ Prolonged sleep deprivation can also induce psychotic symptoms in healthy volunteers, and will occasionally induce severe psychotic reactions with symptoms such as delusions, paranoia and loss of insight.^59–61^

Cannabis users will often cite insomnia as a reason for using the drug.^62^ THC has been investigated as a treatment for sleep problems, though only over the short term.^63,64^ In a study of people seeking treatment for cannabis dependence, sleep problems and strange dreams were reported by more than half of the participants.^65^ Interestingly, even though women experience more withdrawal symptoms overall,^66^ particularly gastrointestinal and mood symptoms, men are more likely than women to report insomnia and vivid dreams during periods of cannabis withdrawal.^67^ This might explain why the association between cannabis use disorder and the development of schizophrenia is stronger in men.^68^ The mechanism through which cannabis dependence and withdrawal disrupted sleep is the subject of ongoing research,^69^ and includes a recent animal model which found that cannabis withdrawal has gender-dependent effects on striatal dopamine release.^70^

A key strength of the present study is the sample size, which is considerably larger than in previous studies of cannabis withdrawal and psychosis. We expanded the available case literature by searching a large database of health records from a population of people with psychosis with a high prevalence of cannabis use. In most of the people identified from this population, the treating clinicians did not identify or describe the presence of cannabis withdrawal. This suggests that there is limited clinical awareness of the syndrome, and that if there had been greater awareness of it, we might have been able to identify more individuals. Consequently, our study probably underestimates the incidence of cannabis withdrawal-associated psychosis: a more accurate measure could be obtained from studies that identify cases prospectively. Another reason that clinicians may have not identified cannabis withdrawal is that many of the symptoms overlap with those which are often observed in acute psychosis. Future studies should therefore collect data on a broad range of cannabis withdrawal symptoms, including those which are not described in the diagnostic criteria, as it may turn out that they have greater diagnostic value in this context. Although our findings provide evidence of an association between cannabis withdrawal and acute psychosis, we were not able to test whether this relationship is causal. A temporal relationship between cannabis withdrawal and psychosis could be coincidental. For example, a person may have stopped using cannabis because their mental state was deteriorating and they were concerned that cannabis use was contributing to this, as has been described in people at clinical high risk for psychosis.^71,72^ We used real-world clinical data to collect symptom data, but because these were collected during routine clinical care and documented in a non-systematic way, some symptoms may have been underreported. Only a minority of the people that we identified (42.6%) had collateral information to confirm that there had been a history of cannabis cessation prior to psychosis onset. In people with psychosis, self-reported estimates of cannabis use and cannabis potency are often inaccurate.^44^ However, there would be little incentive for individuals to disclose recent cessation as well as a history of heavy cannabis use. These limitations meant that our study was not able to estimate the incidence of cannabis withdrawal-associated psychosis.

In conclusion, our findings suggest that, in some individuals, acute psychosis can develop in the context of cannabis withdrawal, and that awareness of the cannabis withdrawal syndrome among clinicians is currently limited. People with psychosis who use cannabis should be advised that it may be safer to reduce their use over time, rather than stopping abruptly. Cannabis withdrawal may also occur after admission to hospital and exacerbate symptoms such as agitation and insomnia. Since cannabis replacement therapy is not available currently, prompt treatment with medications such as benzodiazepines may provide some symptomatic relief.

## Supporting information

Chesney et al. supplementary material Chesney et al. supplementary material

## Data Availability

The analytic code, research materials and data that support the findings of this study may be made available by the corresponding author, though restrictions will apply as they were obtained from a clinical database.
