# Supplementary material for: Psychosis associated with cannabis withdrawal: systematic review and case series
Source: Br J Psychiatry. 2024 Dec 3;226(5):297–308. doi: 10.1192/bjp.2024.175 (PMC7617269; doi:10.1192/bjp.2024.175)
Supplement: Chesney et al. supplementary material [file S0007125024001752sup001.docx]

## Search terms for cannabis withdrawal

abrupt withdrawal $ cannabis

abruptly $ cannabis

abruptly stopped $ cannabis

cannabis cessation

cannabis discontinuation

cannabis withdrawal

cessation $ cannabis

discontinuation $ cannabis

discontinued cannabis

discontinuing $ cannabis

discontinuing cannabis

gave up cannabis $ days

gave up cannabis $ week

quit cannabis $ days

quit cannabis $ week

stopped $ cannabis $ days

stopped $ cannabis $ week

stopped $ cannabis $$ days ago

stopped $ cannabis abruptly

stopped $ cannabis suddenly

sudden withdrawal $ cannabis

suddenly $ cannabis

suddenly stopped $ cannabis

withdrawal $ cannabis

withdrawing $ cannabis

withdrawn $ cannabis

withdrew $ cannabis

## Search terms for psychosis

Bipolar

Mania

Manic

Psychosis

Psychotic

Schizoaffective

Schizophrenia

Schizophrenic

Schizophreniform

## Supplementary Figure 1

Supplementary Figure 1. PRISMA flowchart describing the systematic search
